# Human cardiomyocyte-derived exosomes induce cardiac gene expressions in mesenchymal stromal cells within 3D hyaluronic acid hydrogels and in dose-dependent manner

**DOI:** 10.1007/s10856-020-06474-7

**Published:** 2021-01-19

**Authors:** Burak Derkus

**Affiliations:** Department of Chemistry, Faculty of Science, Ankara University, 06560 Ankara, Turkey

## Abstract

Accomplishing a reliable lineage-specific differentiation of stem cells is vital in tissue engineering applications, however, this need remained unmet. Extracellular nanovesicles (particularly exosomes) have previously been shown to have this potential owing to their rich biochemical content including proteins, nucleic acids and metabolites. In this work, the potential of human cardiomyocytes-derived exosomes to induce in vitro cardiac gene expressions in human mesenchymal stem cells (hMSCs) was evaluated. Cardiac exosomes (CExo) were integrated with hyaluronic acid (HA) hydrogel, which was functionalized with tyramine (HA-Tyr) to enable the development of 3D (three dimensional), robust and bioactive hybrid cell culture construct through oxidative coupling. In HA-Tyr/CExo 3D hybrid hydrogels, hMSCs exhibited good viability and proliferation behaviours. Real time quantitative polymerase chain reaction (RT-qPCR) results demonstrated that cells incubated within HA-Tyr/CExo expressed early cardiac progenitor cell markers (GATA4, Nkx2.5 and Tbx5), but not cTnT, which is expressed in the late stages of cardiac differentiation and development. The expressions of cardiac genes were remarkably increased with increasing CExo concentration, signifying a dose-dependent induction of hMSCs. This report, to some extent, explains the potential of tissue-specific exosomes to induce lineage-specific differentiation. However, the strategy requires further mechanistic explanations so that it can be utilized in translational medicine.

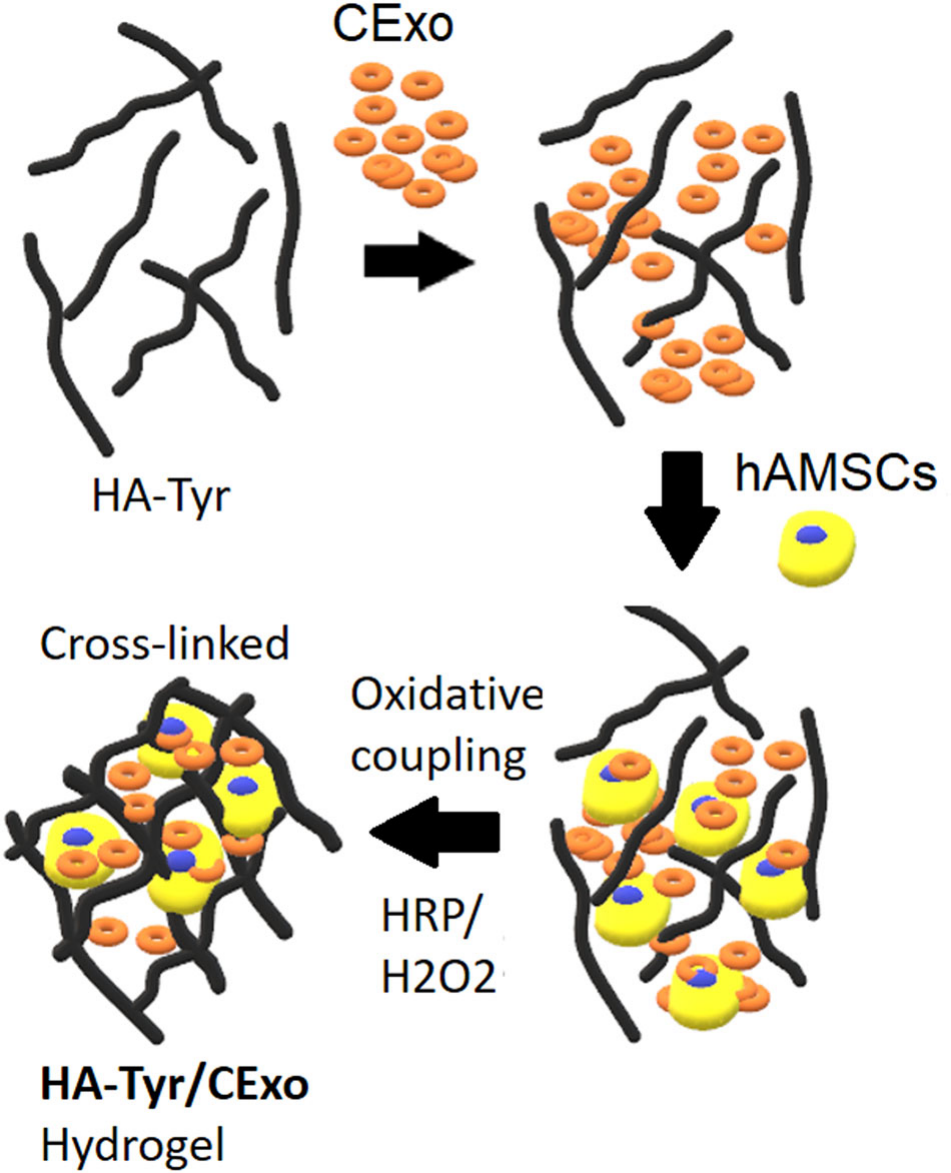

## Introduction

Predicting and controlling lineage-specific differentiation of stem cells, particularly of multipotent and pluripotent cells, is one of the critical issues for tissue engineering and stem cell researches. Cardiogenic differentiation of stem cells is one of the processes in which we have limited knowledge compared with the other cell fates. Cardiogenic differentiation is a dynamic process involving nuclear translocation of NK2 homeobox 5 (Nkx2.5) and GATA Binding Protein 4 (GATA4) [1], the cooperation of Homeobox A10 with Nkx2.5 to regulate the timing of differentiation [2], and the role of micro RNAs (miRNAs) that target different pathways [3].

Currently, different induction approaches to differentiate stem cells into cardiac lineages are being used. 5-Aza-2′-deoxycytidine (5-AZA) is the most extensively used chemical inducer [4] and stimulates both multipotent [5] and pluripotent [6] cells into cardiomyocytes. However, it, induces cells via random demethylation of DNA [5], has carcinogenic potential, which limits in vivo applications [7], and is an exogenous inducer that may cause cell damage. Use of biological inducers including miRNAs [8–10], cytokines such as interleukin-1β, and growth factors namely transforming growth factor-β [11, 12], hepatocyte growth factor, insulin-like growth factor-1, and Islet1 [13, 14] is another strategy for differentiating the stem cells into cardiogenic lineage. Despite these progresses, cardiogenic differentiation remained inefficient (<10%) [15]. Therefore, sophisticated approaches that will improve the yield of differentiation are needed, and it is believed that exosomes can be harnessed for the reliable induction of stem cells.

Exosomes are nano-sized (50–150 nm) vesicles, which tend to be secreted toward extracellular environments [16]. After their rich biological contents including RNAs, miRNAs, proteins, metabolites and roles in biological processes were discovered, exosomes have been used in many fields of biological and medical sciences including cell biology [16], regenerative medicine [17], drug delivery [18] as well as in diagnosis of diseases involving cancer [19], infectious diseases [20] and neurodegenerative diseases [21]. Exosomes also offer an opportunity for cell reprogramming and have been exploited in neurogenic [22], adipogenic [23], osteogenic [24], odontogenic [25] and myogenic [26] differentiation of stem cells. Particularly, exosomes derived from cardiac cells play vital roles in many biological processes. For instance, they modulate mammalian target of rapamycin signalling pathways and attenuate apoptosis [27], manage endogenous stem cell plasticity and tissue regeneration after damage [28] and regulate inflammatory processes [29]. Despite the biological potentials of cardiac exosomes (CExo), their potential in stem cell differentiation has not been explored in vitro. Recent work demonstrated that rat mesenchymal stem cells (rMSCs) could be transformed into cardiomyocytes by co-culturing with cardiomyocytes [30], and this finding reveals that paracrine mechanisms may play a significant role in cardiac trans-differentiation. Inspired by this previous work and those that utilize exosomes for cell reprogramming, the work reported herein seeks to harness the potential of exosomes for lineage-specific differentiation—cardiac trans-differentiation—of mesenchymal stem cells.

To demonstrate the possibility to use exosomes to induce lineage-specific differentiation, three-dimensional (3D) hyaluronic acid (HA)-based hydrogels encapsulating human cardiomyocyte cells-derived exosomes were developed. HA was used to provide a biocompatible microenvironment, which was functionalized with Tyr (HA-Tyr) to control stability and mechanical properties through orthogonal enzymatic cross-linking reaction of Tyr that results in the formation of dityramine moiety, as previously described by Derkus et al. [31]. It brings various advantages in 3D cell culture, such as fast gelling time (<1 min), gradually degradation by hyaluronidase enzyme, and ease-of-manipulation. Besides, HA is the largest glycosaminoglycan macromolecule in cardiac ECM [32] and involved in cardiac development [33]. All these features make HA-based hydrogels one of the most suitable materials for cardiac-cell-culture applications. The strategy proposed in this work opens opportunities for growth-factor-free induction of human mesenchymal stem cells (hMSCs) into cardiac cells and in vivo application for cardiac tissue regeneration thereof.

## Materials and methods

### hMSCs and human cardiomyocytes cell culture

The hMSCs (derived from healthy human adipose tissue) were supplied by Dr. Yavuz Emre Arslan’s laboratory (Canakkale On Sekiz Mart University, Turkey). The MSCs were in spindle-shape fibroblastic morphology and expanded using a commercial low-serum hMSC growth medium (Cat No. 7501, ScienCell, USA). Passages 4–6 were used for the experiments. Human cardiomyocyte (HCM) cells isolated from the ventricles of healthy adult human heart (PromoCell, Germany) were cultured in high glucose Dulbecco’s Modified Eagle’s medium (Sigma-Aldrich Inc., USA) supplemented with 10% exosome-depleted foetal bovine serum (System Biosciences, USA) and 1% penicillin/streptomycin (Thermo Fisher Scientific, USA). The cells were detached using Trypsin-ethylenediaminetetraacetic acid (Thermo Fisher Scientific, USA) when they reached confluency.

### Isolation and characterisation of CExo

Exosomes were isolated from primary human cardiomyocyte cells via serial ultracentrifugation as previously described [19]. Briefly, conditioned medium (CM) was collected from primary human cardiomyocyte cells that were cultured at 37 °C under 5% CO_2_ conditions. CM was then centrifuged (Universal 320 R, Hettich, Germany) for 15 min at 300 *g* and 4 °C to remove the cells, filtered through 0.22-μm filters, centrifuged again for 15 min at 10,000 *g* and 4 °C—to remove cell remnants, apoptotic bodies and microvesicles—and finally ultracentrifuged (Himac CP100WX, Hitachi, USA) for 70 min at 120,000 g and 4 °C to pellet the exosomes. The isolated exosomes were re-suspended in medium and characterized with a bicinchoninic acid (BCA) test kit (Thermo Fisher Scientific, USA), ELISA kit for CD63 (System Bioscience, USA) and transmission electron microscopy (TEM).

For the quantification of exosomal protein content, exosome lysates were obtained by adding an equal volume of the radioimmunoprecipitation assay buffer (Thermo Fisher Scientific, USA) including protease inhibitors. Protein standards were prepared using bovine serum albumin in concentrations between 5 μg mL^−1^ and 50 mg mL^−^^1^. Optic measurements of protein standards and exosome samples were recorded at 562 nm on a microplate reader (Thermo Scientific, Multiskan Sky Microplate Spectrophotometer) after incubation with BCA reagent at 37 °C for 30 min. The level of CD63+ exosome particles was quantified with ExoELISA CD63 (System Biosciences) following the user instruction. TEM, additionally, was used to confirm the morphology of the individual exosomes and their specific diameter changing between 50 and 150 nm. For this purpose, 10 µL of sample was treated with 4% paraformaldehyde for fixation and 10 μL of the fixed exosomes were deposited onto a piece of parafilm. TEM grid was laid down onto droplet, and allowed to adsorb exosomes for 10 min. The grids were transferred onto a 1% glutaraldehyde droplet placed onto parafilm for further fixation. After counterstaining with 0.5% uranyl acetate for 2 min, exosomes were observed under TEM (JEM 2100-F, USA).

### Development of CExo loaded HA-Tyr (HA-Tyr/CExo) 3D hybrid gels

Stable 3D hydrogels comprising HA-Tyr (obtained from Creativepegworks, Cat No: HA-387, MW 2.500 k), CExo and hMSCs were formed by mixing CExo with HA-Tyr, inoculating the cells and triggering oxidative coupling reaction via interaction of hydrogen peroxide (H_2_O_2_) and horse radish peroxidase (HRP) (Fig. S1). The gels to be tested in experimental setups were prepared in 96-well plates as follow: 50-μL HA-Tyr—that contains HRP (3 U/mL)—in different concentrations (final conc. 1, 3 and 6%) was placed in wells, and CExo in different concentrations (10, 50 or 100 μg in 5, 10 and 20 μL medium, respectively) were added thereafter. The gelation was triggered by injecting 5-μL H_2_O_2_ (1-mM Stock, 0.0125 mM final conc.) following cell inoculation in 5-μL medium. HA-Tyr works through a very fast gelling mechanism, which is <1 min, so the gels could be washed for several times right after the cross-linking step, to prevented cell damage. This very fast gelling property makes HA-Tyr a suitable material for 3D cell culture.

### Microstructural and mechanical characterisations of HA-Tyr gels

The micro-architecture and mechanical properties of HA-Tyr gels in varying mechanical strength were examined by monitoring with scanning electron microscopy (SEM) as well as testing with the micro-mechanical tester. Gels were processed for SEM with freeze-dryer to remove the water, then sputtered with gold at 20 mA for 45 s, and examined under SEM (FEI 430 Nova NanoSEM, USA). In addition, compression test was performed on cylindrical hydrogels with 8-mm diameter and 5-mm heigth to determine the mechanical strength of 1, 3 and 6% HA-Tyr hydrogels. The analysis was performed at 2-mm/min loading rate and under a load of 5 N (Cellscale, Canada). Young’s moduli were calculated based on the stress/strain curves. All the measurements were performed in duplicate.

### CExo release study from HA-Tyr/CExo gels

To assess the potential of the HA-Tyr/CExo as a cell induction scaffold, CExo release kinetics was investigated. For this purpose, HA-Tyr/CExo hydrogels containing 1-mg CExo were prepared and incubated in cell culture media in an incubator at 37 °C for different time points (30 min, 1 h, 3 h, 6 h, 1 d, 3 d). BCA assay was applied to the 100 μL of supernatant of each gel to measure exosomal protein content after each time point. Each time point was studied triplicate.

### Investigation of cell viability utilizing the Live–Dead assay and MTS test

Suitability of HA-Tyr/CExo for cell culture applications and effects of different parameters such as gel mechanical property and CExo concentration on cell survival and proliferation were investigated with calcein/ethidium homodimer-1 (ethd-1) staining and MTS (3-(4,5-dimethylthiazol-2-yl)-5-(3-carboxymethoxyphenyl)-2-(4-sulfophenyl)-2H-tetrazolium) testing. For a live–dead assay, hMSCs (5000 cells/gel) were seeded in HA-Tyr/CExo and on tissue culture plastic (TCP, positive control), cultured for 1 and 7 days, and stained with calcein (4 μM) and ethd-1 (2 μM) (Molecular Probes, Thermo Fisher, UK) in the dark for 30 min at 37 °C. The cells were monitored under fluorescence microscope (Leica DMIL, Germany) at 488 and 527-nm excitation wavelengths for calcein and ethd-1, respectively.

Cell proliferation was also tested using the MTS kit (Cell Titre, Promega, USA), unlikely the cell density of 7500 cells/hydrogel in this case. After definite time points (1, 3, and 7 days), the waste medium was discarded, and 20 μL of MTS reagent was added in each well containing 100 μL of fresh culture medium. After 1 h incubation in the dark at 37 °C, absorbance values for HA-Tyr (1, 3, 6%), HA-Tyr/CExo, that comprise varying concentrations of exosomes (10, 50, 100 μg), and TCP (positive control) groups were recorded at 490 nm. Each parameter was studied triplicate. Tukey’s multiple comparison test was used for the statistical evolution and comparison of groups including hydrogels in different concentrations, and hydrogels with different CExo concentrations.

### Investigation of cardiac gene expressions

Cardiac gene expressions by hMSCs that were cultured within HA-Tyr and HA-Tyr/CExo hydrogels were investigated with RT-qPCR. To this end, total RNA was extracted from hydrogels using a total RNA isolation kit (GeneDireX, USA), quantified with a spectrophotometer (Thermo Scientific, Multiskan Sky Microplate Spectrophotometer), and a total of 120 ng RNA was reverse-transcribed using iScript cDNA Synthesis Kit (Bio-Rad, USA). For RT-qPCR, each reaction was set duplicate, using SYBR green mastermix and performed in a Bio-Rad CFX96 instrument. Results were normalized to the reference gene (GAPDH), and the statistical differences in gene expressions were evaluated using a two-way analysis of variance test. Primer sequences used in this study were listed in Table S1.

## Results

### Characterisation of CExo

In order to characterize exosomes isolated with ultracentrifugation method, three different characterization techniques namely TEM, ELISA test and BCA test were applied. TEM micrograph confirmed that isolated vesicles were in spherical morphology, having a size around 50 nm (Fig. S2a). Besides, the number of CD63+ exosome particles was found to be 1.17 × 10^10^ (optic density: 0.62; calibration graph has been presented in Fig. S2b), which further proved the success of exosome isolation. Exosome concentration was calculated using the BCA test in terms of total protein content of CExo. Utilizing the calibration equation (presented in Fig. S3) and using the absorbance obtained by CExo (optic density: 1.52), CExo concentration was found to be 3.7 mg mL^−1^.

### Micro-scale investigation and mechanical properties of HA-Tyr and HA-Tyr/CExo hydrogels

**Fig. 1:**
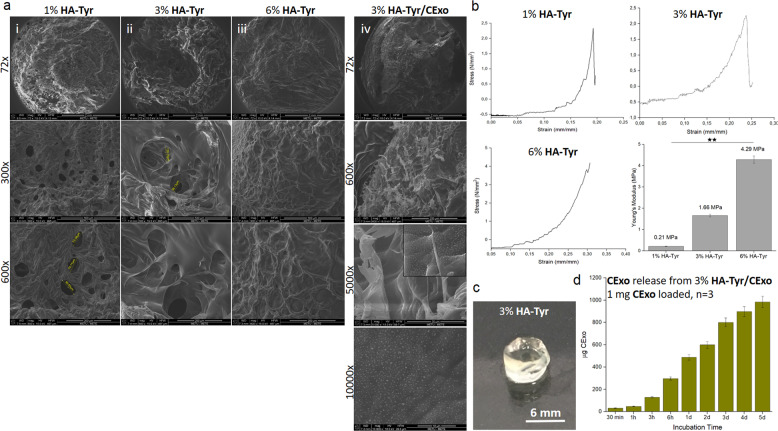
Characterization of HA-Tyr and HA-Tyr/CExo hybrid hydrogels: microstructural characterization—using SEM—of HA-Tyr hydrogels in different stiffness as well as HA-Tyr/CExo hybrid hydrogel (**a**). Stress–strain curves related to compression test of 1, 3 and 6% HA-Tyr (**b**). Macro-view and 3D-view of HA-Tyr-based hydrogels (**c**). CExo release kinetics from 3% HA-Tyr/CExo hydrogels (**d**) (**p < 0.01)

SEM observations revealed that the HA-Tyr hydrogels exhibited a whole and microporous morphology (Fig. 1a (i–iii)), as previously demonstrated [34]. In addition, average pore sizes for each gel were calculated using imageJ software (National Institute of Health) considering ten pores per SEM image (three SEM images were used for each gel concentration) and found to be 75.57 ± 5.20, 69.20 ± 4.80 and <50 μm for 1, 3 and 6% HA-Tyr, respectively. Expectedly, it was observed that the pore diameter decreased slightly with increasing gel concentration. In the HA-Tyr/CExo hydrogel, the exosomes were seen to integrated with HA-Tyr and were evenly and individually distributed throughout the gel (Fig. 1a (iv)).

The main goal of this design was to develop a bioactive and robust hydrogel that could trigger a particular process. To assess the mechanical stability of the hydrogels, which is essential to reveal the gel response and failure, compression test was applied in 1, 3 and 6% HA-Tyr. The compressive moduli of HA-Tyr hydrogels with different concentrations (1, 3 and 6%) were obtained from the linear region of typical stress–strain curves (Fig. 1b). The Young’s moduli of 1, 3, and 6% HA-Tyr (*n* = 2) were found to be 0.21, 1.66 and 4.29 MPa, respectively, which were seen to increase with ascending HA-Tyr concentration. In addition, HA-Tyr-based gels were seen to have transparency (Fig. 1c), that is important in cell culture applications—particularly in microscopic observations.

### HA-Tyr-based gels release CExo in a timely manner

Optimal release kinetics is a crucial parameter for efficient induction of the encapsulated cells as well as promoting the repair of damaged issue. Therefore, it was tested whether HA-Tyr/CExo hydrogel could effectively retain exosomes inside or it released immediately after being treated with the medium. HA-Tyr/CExo hydrogel retained almost half of the CExo within the gel in the first 2 days, while the cumulative release of CExo was found until nearly 5 days (Fig. 1d).

### hMSCs survive and proliferate in 3D HA-Tyr and HA-Tyr/CExo hybrid hydrogels

**Fig. 2:**
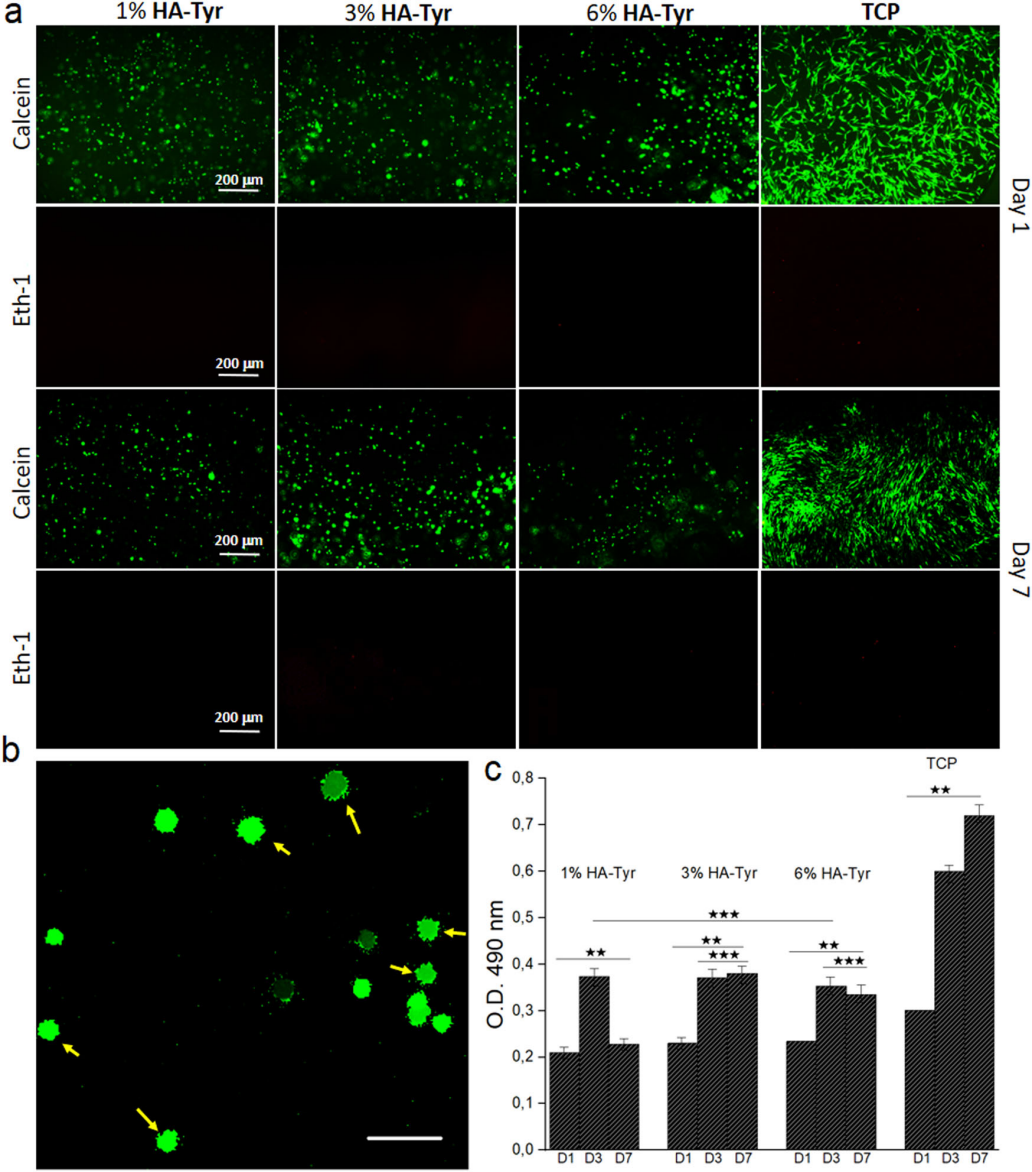
Cell viability and proliferation study: fluorescence images obtained with calcein and Ethd-1 for hMSCs cultured within HA-Tyr hydrogels with different stiffness (**a**). Confocal image of hMSCs within 3% HA-Tyr showing the slight cell attachment and spreading. Scale bar 50 μm (**b**). MTS testing results for hMSCs cultured within 1, 3 and 6% HA-Tyr for 1, 3 and 7 days (**c**) (**p < 0.01, ***p > 0.05)

To investigate the pertinence of the material as a cell culture construct, live/dead imaging with calcein and ethd-1 in order to monitor the viability of the cells in addition to MTS tests were applied. As expected, HA-Tyr did show good cytocompatibility regardless of concentrations (Fig. 2a). However, 3% HA-Tyr did show better cell survival after 7 days, with a significantly higher number of viable cells compared to 1 and 6% HA-Tyr. This might be attributed to the optimal mechanical sensing by the cells for them to slightly attach to the HA-Tyr (Fig. 2b), which is also an important process for cell differentiation. On the other hand, almost no dead cells were observed. To further examine the potential of the hydrogels for cell proliferation, MTS test was conducted for 1, 3 and 6% HA-Tyr. Cell numbers were increased from day 1 to 7 within 3% HA-Tyr, remained stable after day 3 within 6% HA-Tyr, while it decreased after day 3 within 1% HA-Tyr (Fig. 2c).

**Fig. 3:**
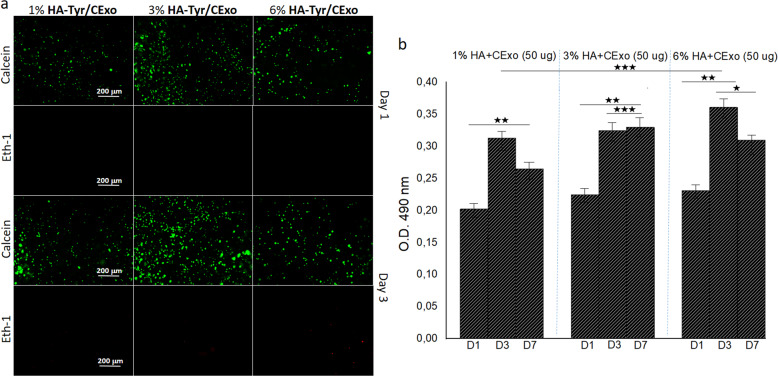
Cell viability and proliferation study for hybrid hydrogels: fluorescence images obtained with calcein and Ethd-1 for hMSCs cultured within HA-Tyr/CExo hydrogels with different stiffness (**a**). MTS testing results for hMSCs cultured within 1, 3 and 6% HA-Tyr/CExo (50 μg) for 1, 3 and 7 days (**b**) (*p < 0.05, **p < 0.01, ***p > 0.05)

In order to reveal the effects of CExo on cell viability and proliferation, these experiments were repeated for 1, 3 and 6% HA-Tyr/CExo hydrogels that bear 50-μg CExo. Fluorescence images obtained with calcein and ethd-1 staining showed no toxicity—dead cells—and good viability at days 1 and 3 (Fig. 3a). Amongst the gels, 3% HA-Tyr/CExo came to the forefront with slightly higher cell viability. MTS results confirmed these observations with a similar fashion in HA-Tyr only hydrogels (Fig. 3b). Therefore, 3% HA-Tyr/CExo was used throughout the work.

**Fig. 4:**
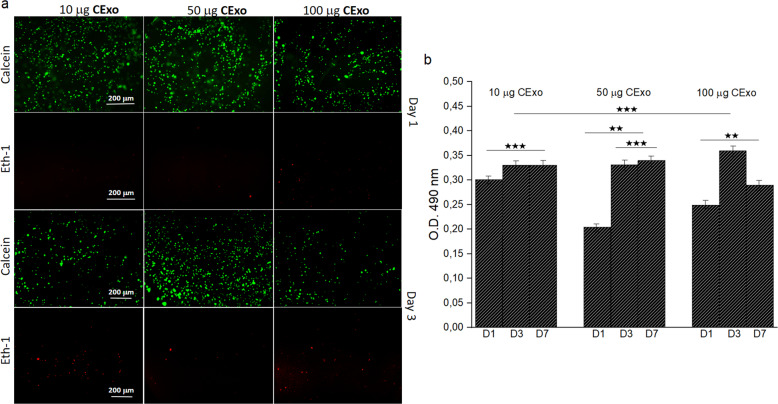
Investigation of effects of CExo on cell viability and proliferation: live–dead assay images of hMSCs cultured within 3% HA-Tyr/CExo hydrogels that contain varying concentrations of CExo (10, 50 and 100 μg) (**a**). MTS testing results for hMSCs cultured within 3% HA-Tyr/CExo (10, 50 and 100 μg) for 1, 3 and 7 days (**b**) (**p < 0.01, ***p > 0.05)

Possible toxic effects of CExo on hMSCs were assessed by treating the cells with varying levels of exosomes. For this purpose, hMSCs were cultured within 3% HA-Tyr/CExo hydrogels that contain 10, 50 and 100-μg CExo. Live/dead assay revealed a similar cell viability image for all the gels with a little difference in favour of 3% HA-Tyr/CExo loaded with 50-μg CExo (Fig. 4a). Unlikely HA-Tyr-only gels, there seemed some dead cells in HA-Tyr/CExo hydrogels, which was minimum in 3% HA-Tyr/CExo loaded with 50-μg CExo. MTS testing further supported live/dead assay results and indicated an insignificant difference between groups (Fig. 4b).

### CExo differentiate hMSCs into early cardiac progenitor cells and induce cardiac gene expressions

**Fig. 5:**
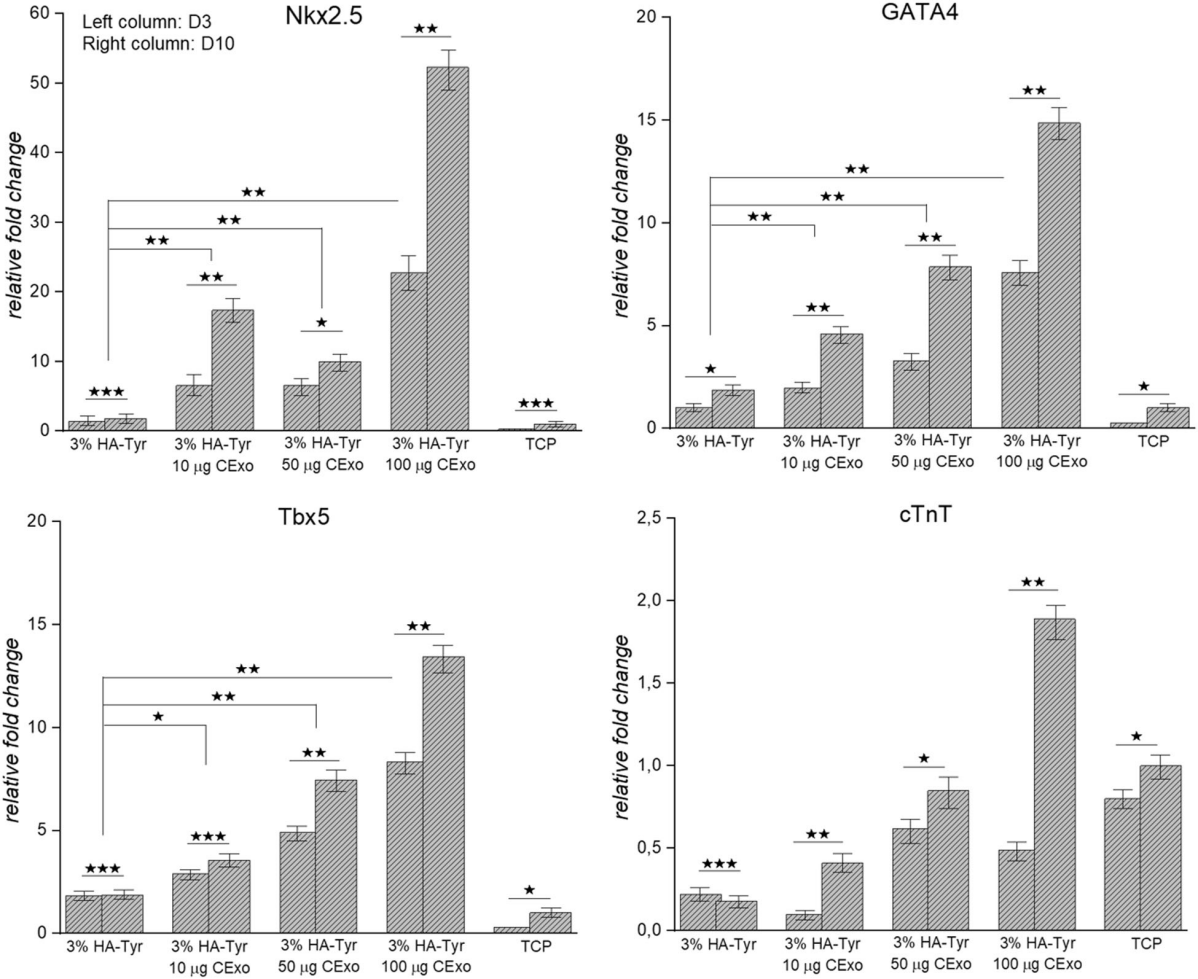
Gene expression study: expressions of cardiac genes (Nkx2, GATA4, Tbx5, cTnT) in 3% HA-Tyr/CExo (10, 50 and 100 μg) hydrogels after 3 (left column) and 10 (right column) days of induction (*p < 0.05, **p < 0.01, ***p > 0.05)

The goal of this study was to design robust, biocompatible and bioactive hydrogel that is able to induce hMSCs toward a cardiogenic lineage benefiting from natural inducers instead of chemicals or growth factors. To prove the hypothesis (Fig. S1b), hMSCs were exposed to CExo within 3% HA-Tyr/CExo (10, 50 and 100 μg) hydrogels for 3 and 10 days, and expressions of cardiac genes—GATA4, T-box transcription factor (Tbx5), Nkx2.5 and cardiac troponin T (cTnT)—were investigated by RT-qPCR. The cells cultured in 3% HA-Tyr/CExo hydrogels were seen to begin expressing early cardiac progenitor markers, GATA4, Tbx5, Nkx2.5, after 3 days’ induction (Fig. 5). These expressions significantly increased at day 10, which were almost doubled compared to day 3. The cells cultured within 3% HA-Tyr and on TCP did not exhibit this behaviour. On the other hand, the expression of cTnT by the cells inoculated in HA-Tyr/CExo hydrogels was not significant. Though the expression of cTnT by the cells within 3% HA-Tyr/CExo (100 μg) was nearly four times higher at day 10 compared to day 3, the cumulative expression of these gene was below twofolds compared to cells cultured within 3% HA-Tyr and on TCP (negative controls in this case).

## Discussion

In this study, it was intended to harness the biological potential of tissue-specific cells-derived exosomes for the induction of hMSCs into cardiac-like cells. For this, 3D cell culture construct consisting of HA-Tyr as scaffolding material and CExo as biological elements was developed.

HA-Tyr/CExo hydrogel was thoroughly characterized with SEM, micro-mechanical analysis, and by examining exosome-release kinetics. In addition, live/dead assay and MTS testing were applied so as to reveal the potential for cell growth and proliferation. SEM observation suggested that exosomes were successfully encapsulated inside the hydrogel, and covered the pores and flakes such that they combined and retained all the features of HA-Tyr and exosomes (Fig. 1a). The obtained hydrogels were extremely strong and tough—as well as transparent—(Fig. 1b, c), of which mechanical strength can be tuned by adjusting the concentration. The obtained release profile further demonstrated that exosomes were effectively encapsulated in the hydrogel, and exhibited a sustained release behaviour (Fig. 1d). This property is essential for cell induction in vitro/vivo by durably exerting the potential of exosomes and is expected to implement defect regeneration when applied in vivo. Similar release kinetics of exosomes were reported previously [35, 36].

Live/dead assay and MTS testing revealed that 3% HA-Tyr was a suitable candidate for hMSCs survival (Fig. 2). A decrease in cell number within 1% HA-Tyr after day 3 could be explained with the poor mechanical strength of the material, which resulted in cell leakage. Considering the live/dead assay and MTS testing results, it can be concluded that HA-Tyr concentration does not affect cell viability/proliferation in the short term, but it does at the long-term culture. In addition, HA-Tyr hydrogels are nontoxic and provide a suitable environment for cell culture applications. Considering the better cell viability and proliferation, in addition to moderate mechanical strength, which is important for the target application—soft tissue—[37, 38], 3% HA-Tyr/CExo was selected and used throughout the work. In addition, the live/dead assay and MTS testing showed that CExo had no toxic effects on hMSCs regardless of the concentration (Figs 3 and 4). These findings were in accordance with literature works [39, 40], and demonstrated that exosomes can be safely used in biological applications.

Cardiac-cell-derived exosomes were shown to increase cardiac-related gene (Nkx2.5, GATA4 and Tbx5) expressions in hMSCs (Fig. 5). However, the increase in cTnT was not significant. This low-level expression of cTnT compared to other cardiac genes was indeed meaningful in as much as this gene is expressed in a mature form of cardiac cells—cardiomyocytes—that come out in the later stages of cardiogenesis [41, 42]. Similar findings have previously been reported [43]. Another striking finding in gene expression study was that 3% HA-Tyr/CExo hydrogels induced cardiac gene expressions in hMSCs in a dose-dependent manner (Fig. 5). Expressions in GATA4 and Tbx5 increased with ascending exosome levels, from 10 to 100 μg. That is, cardiac gene expressions can be tuned by changing CExo concentration.

## Conclusion

In this proof-of-the-concept study, exosomes derived from cardiac cells were combined with HA-based hydrogels, of which cardiogenic induction potential was then tested. This strategy harnessing the biological potentials of exosomes could be effectively utilized in inducing cardiac gene expressions in hMSCs; however, it did not ensure a full differentiation of the cells into functional cardiomyocytes. Instead, it is extrapolated and assumed that GATA4+/Nkx2.5+/Tbx5+/cTnT-early cardiac progenitors were formed. These findings will be validated further in the future using histological techniques as well as mechanistic approaches such as transcript profiling and proteomics. This strategy is anticipated to have an enormous effect on the regeneration of damaged tissues in the coming years.

## Supplementary information

Supplementary Information
