# Supplementary material for: Human cardiomyocyte-derived exosomes induce cardiac gene expressions in mesenchymal stromal cells within 3D hyaluronic acid hydrogels and in dose-dependent manner
Source: J Mater Sci Mater Med. 2021 Jan 19;32(1):2. doi: 10.1007/s10856-020-06474-7 (PMC7815535; doi:10.1007/s10856-020-06474-7)
Supplement: Supplementary file 1 — Supplementary Information [file 10856_2020_6474_MOESM1_ESM.docx]

Human Cardiomyocyte-Derived Exosomes Induce Cardiac Gene Expressions in Mesenchymal Stromal Cells within 3D Hyaluronic Acid Hydrogels and in Dose-Dependent Manner

Burak Derkus

Department of Chemistry, Faculty of Science, Ankara University, 06560 Ankara, Turkey

[burakderkus@gmail.com](mailto:burakderkus@gmail.com)

Mobile: +905069069786

**SUPPLEMENTARY INFORMATION**

**
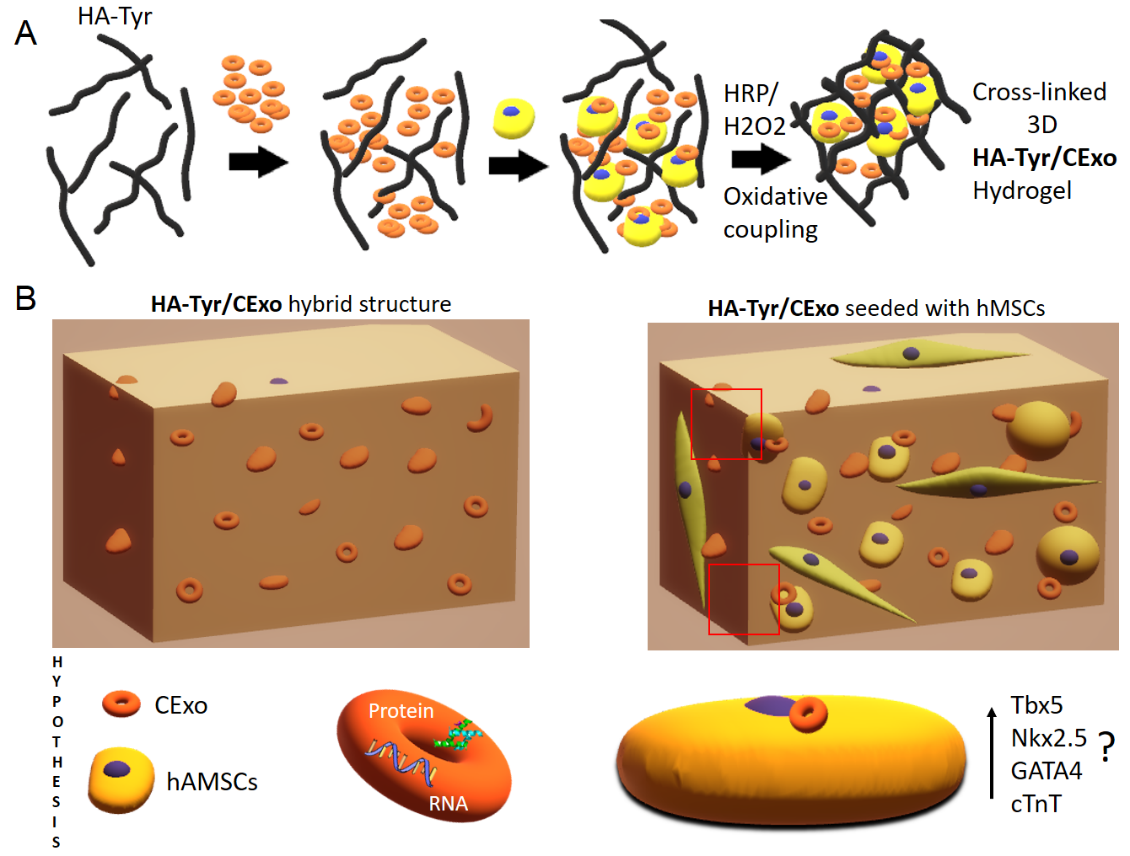
**

**Figure S1 –** Schematic illustration of hydrogelation/cross-linking mechanism of HA-Tyr after inoculation of exosomes and stem cells (A). 3D view of HA-Tyr/CExo w/wo hAMSCs (B). Below illustration shows the hypothesis/strategy of this work.


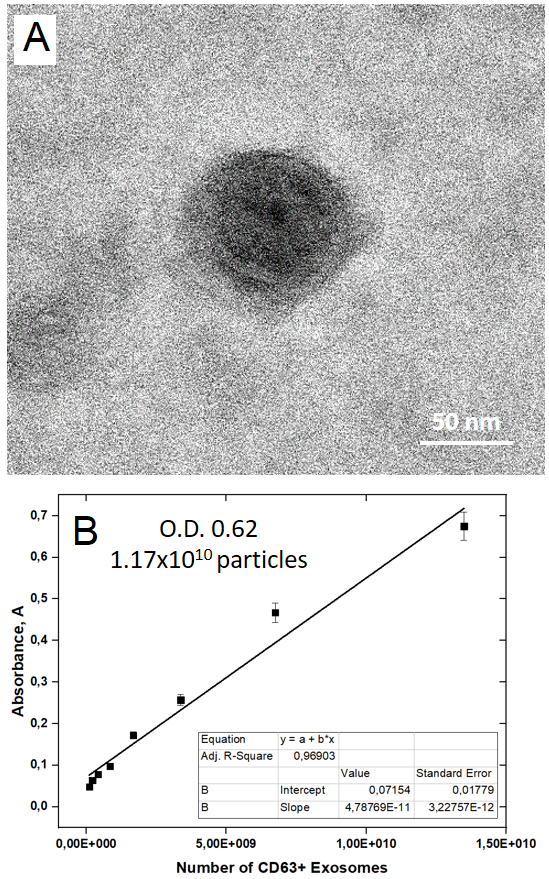


**Figure S2 –** Characterization of cardiac exosomes: TEM image of CExo (A). Calibration graph related to ELISA test against CD63 antibody, an exosomal biomarker (B).


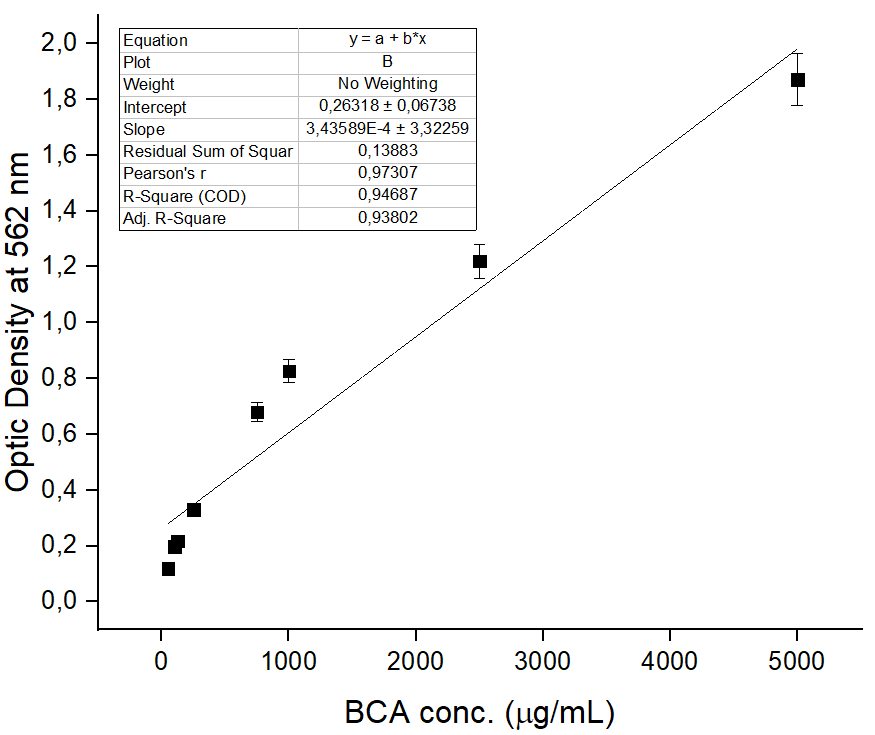


**Figure S3 –** Calibration graph related to BCA test

**Table S1.** Primer sequences used in qPCR study

| Genes | Primer, Forward | Primer, Reverse |
| --- | --- | --- |
| cTnT | GGCAGCGGAAGAGGATGCTGA | GAGGCACCAAGTTGGGCATGAACGA |
| GATA4 | CTGTGCCAACTGCCACACCA | GGCTGACCGAAGATGCGTAG |
| Nkx2.5 | CTGCCGCCGCCAACAAC | CGCGGGTCCCTTCCCTACCA |
| Tbx5 | AGTCCCCCGGAACAACTCGAT | ACAGCAGCTGCACCGTCACC |
